# Effects of Sport Climbing on Multiple Sclerosis

**DOI:** 10.3389/fphys.2017.01021

**Published:** 2017-12-19

**Authors:** Julia Steimer, Robert Weissert

**Affiliations:** Department of Neurology, University of Regensburg, Regensburg, Germany

**Keywords:** sport effects, sport climbing, physical activity, exercise, multiple sclerosis, central nervous system

## Abstract

Multiple sclerosis (MS) is an autoimmune and neurodegenerative disease of the central nervous system (CNS) with different types of disease courses (relapsing-remitting, secondary-progressive, primary progressive) that leads to physical as well as mental disability. The symptoms comprise paresis or/and paralysis, ataxia, bladder dysfunction, visual problems as well as effects on cognition. There is limited data regarding the possible effects of sport climbing respectively therapeutic climbing on patients with MS. Sport climbing offers many potentially beneficial effects for patients with MS since there are effects on coordination, muscular strength, and cognition to name the most relevant ones. Also, disease models in rodents point toward such positive outcomes of climbing. Therefore, we assessed the currently available research literature on general effects of physical exercise, impact of climbing on body and mind and therapeutic climbing for prevention or therapy for the treatment of MS. The sparse published controlled trials that investigated this sport activity on different groups of patients with neurological or geriatric diseases grossly differ in study design and outcome parameters. Nevertheless, it appears that climbing offers the opportunity to improve some of the symptoms of patients with MS and can contribute to an enhanced quality of life.

## Introduction

Multiple sclerosis (MS) is one of the most frequent organ specific diseases of the central nervous system (CNS) The etiology of MS is still not fully explored. Beside autoimmune and degenerative processes, genetic factors also contribute to the disease (Riedhammer and Weissert, 2015). MS leads to demyelination and axonal loss and predominantly affects the white but also the gray matter of the CNS (Granberg et al., 2017). Due to damages in the myelin sheath, nerve conduction is disturbed (Wang et al., 2015). Axonal loss is a major component leading to disability. Depending on the localization of the lesions the symptoms differ strongly between affected patients. Many symptoms can emerge during the course of the disease (http://www.nationalmssociety.org/Symptoms-Diagnosis/MS-Symptoms; Kesselring and Beer, 2005; Table 1). Patients with MS also suffer from restrictions regarding occupational and social life. With varying degree of severity of the symptoms the personal resilience can be strongly reduced which can lead to increased social isolation and a complete inability to carry on work (Incerti et al., 2017). MS is a chronic life-long disease that cannot be cured up to date (Weissert, 2013). The treatment options for MS can be divided in therapeutic approaches aiming at treatment of an acute bout (relapse) and prevention of further relapses as well as reduction of disability progression by disease modifying therapeutics (Weissert, 2013). Modern immunomodulatory therapy can achieve a disease-free status in several patients with MS (Giovannoni et al., 2017). Additional therapeutic options include symptomatic medicinal therapy as well as physical and occupational therapy (Kesselring and Beer, 2005). For the alleviation of fatigue, activity methods such as endurance and balance training are recommended (Heine et al., 2015). Other treatment options for fatigue appear to be mostly pharmacological but without good outcome (Braley and Chervin, 2010). Since rehabilitation methods have mainly been applied to patients in an advanced stage of the disease the question whether an early physical intervention in combination with common drug therapy may contribute to prevention of long-term disability remains partly unresolved (Kesselring and Beer, 2005; Motl and Pilutti, 2012; Khan et al., 2017).

**Table 1:** Symptoms of MS patients and possibly interference by sport activity based on outcome of published studies and reviewed literature (Kesselring and Beer, 2005; Motl and Pilutti, 2012).

**Symptoms**	**Interference by sports**
Optic neuritis	Not likely (–)
Oculomotor symptoms	Not likely (–)
Cranial nerve palsies	Not likely (–)
Paresis	Likely (+) (Zamparo and Pagliaro, 1998; Rodgers et al., 1999; Sandroff et al., 2014; Davies et al., 2016)
Sensory symptoms	Likely (+)
Cerebellar symptoms (mainly ataxia)	Likely (+) (Rodgers et al., 1999; Keller and Bastian, 2014; Davies et al., 2016)
Bladder dysfunction	Not likely (–)
Cognitive impairments	Likely (+) (Velikonja et al., 2010; Sandroff et al., 2014; Zimmer et al., 2017)
Fatigue	Likely (+) (Oken et al., 2004; Velikonja et al., 2010; Heine et al., 2015)
Psychological changes	Likely (+) (Heine et al., 2015)

Sport climbing becomes more and more popular and evolves into a trend sport (Buechter and Fechtelpeter, 2011). Lately, also the therapeutic potential of climbing for various disease conditions has been discovered. Because it entails a comprehensive involvement of different bodily functions, climbing can be used for the prevention, treatment and deceleration of progression of various diseases or disabilities. It appears to be a medically sensible intervention, especially in patients suffering from disease-associated neurological impairments (Kern, 2010; Grzybowski and Eils, 2011). Therapeutic climbing is constructed of sequences of movement comparable to sport climbing and requires similar basic prerequisites. Therefore, we assessed published data reporting the impact of a climbing program on different health conditions with focus on multiple sclerosis (MS).

## Methods

### Literature search and identification of studies

PubMed and the Web of Sciences were searched with the aid of the terms “indoor climbing,” “rock climbing,” “therapeutic climbing,” and “bouldering” accompanied by the Boolean operator “AND” and “multiple sclerosis” and “neurological disease.” After scanning the abstracts, the relevant full-texts were selected for further review. Additional literature was extracted through examination of the bibliography of the selected full-texts. Trials which have a focus on the effects of a climbing intervention on diverse physical conditions were retained. In nine of the selected trials, the intervention is described as “sport climbing,” “indoor climbing,” “therapeutic climbing,” or “bouldering” (Mazzoni et al., 2009; Fleissner et al., 2010; Velikonja et al., 2010; Engbert and Weber, 2011; Stephan et al., 2011; Kim and Seo, 2015; Luttenberger et al., 2015; Aras and Ewert, 2016; Schram Christensen et al., 2017). The other study approaches the effects of a tightrope course on patients in psychotherapeutic treatment (Mehl and Wolf, 2008). Even if a high wire intervention is not really climbing in the narrower sense, the outcomes of the study were considered due to similarities between these two types of exercises. The selected trials are summarized in Table 2.

**Table 2:** Studies containing a climbing intervention.

**Author**	**Name of the study**	**Year**	**Number of Participants**	**Condition**	**Durance of the study**	**Frequency of the intervention**	**Total time of intervention**	**Type of the intervention**	**Important results**
Mehl and Wolf	Experiential learning in psychotherapy. Evaluation of psychophysical exposure to a tightrope course as adjunct to inpatient psychotherapeutic treatment	2007	247 (155 persons in the intervention group, 92 persons in the control group)	Patients with mental disorders	Not mentioned	Average of 2 visits in the tightrope course by each participant	Not mentioned	Tightrope course (height up to 12 m)	Greater improvement in some outcome criteria (for example depression, self-efficacy, self-rated quality of life, general psychological impairment)
Mazzoni et al.	Effect of indoor wall climbing on self-efficacy and self-perceptions of children with special needs	2009	46 (23 children in the intervention group an 23 in the control group)	6 to 12 year old children with special needs	6 weeks	1 × 1 h/week	6 h	Indoor climbing	Improvement of self-efficacy
Fleissner et al.	Therapeutic climbing improves independence, mobility and balance in geriatric patients	2010	95 (48 persons in the intervention group; 47 persons in the control group)	Patients from geriatric ward	Not mentioned	5 × 30 min	2.5 h	Therapeutic climbing (wall up to 2.90 m); Conventional physiotherapy for the control group	Handgrip strength, Tinetti-test, timed up & go test and ADL (activity of daily living, Barthel-Index) ↑ in both groups; Number of falls ↓ in both groups; Significant improvements by therapeutic climbing with respect to the timed up & go test, the Tinetti test and ADL in comparison to the control group
Velikonja et al.	Influence of sport climbing and yoga on spasticity, cognitive function, mood and fatigue in patients with multiple sclerosis	2010	20	Relapsing-remitting MS or progressive MS, EDSS < 6, EDSS pyramidal functions score >2	10 weeks	1/week	Not mentioned	Sport climbing (wall up to 5 m, inclination 90°) and yoga	Decline in EDSS pyramidal functions score in both groups; improvement of selective attention in the yoga-group; reduced fatigue in the climbing-group
Stephan et al.	Effect of long term climbing training on cerebellar ataxia: a case series	2011	4	Cerebellar ataxia	6 weeks	From 2 × 30 min/week to 3 × 60 min/week depending on the individual physical condition	9–18 h for each participant	Indoor climbing (wall up to 2.5 m, inclination adjustable)	Movement velocity ↑; Balance ↑ in 2 patients; Manual dexterity ↑ in 2 patients
Engbert and Weber	The effects of therapeutic climbing in patients with chronic low back pain	2011	28 (14 persons in the intervention group, 14 persons in the control group)	Patients with chronic low back pain	4 weeks	4 × 45 min/week	12 h	Therapeutic climbing (wall up to 2.5 m); standard exercise therapy for the control group	Significant improvements in 3/8 subscales of the SF-36 for both groups; In 2/8 subscales, only the therapeutic climbing group improved; In 1/8 subscales only the control group improved
Kim and Seo	Effects of a therapeutic climbing program on muscle activation and SF-36 scores of patients with lower back pain	2015	30 (15 persons in the intervention group and 15 persons in the control group)	Patients with chronic lower back pain	4 weeks	3 × 30 min/week	6 h	Therapeutic climbing (wall up to 3 m, inclination 90°); Lumbar-stability mat exercises for the control group	SF-36 score (health related quality of life)↑ in both groups; surface-electromyography-activities ↑ in both groups
Luttenberger et al.	Indoor rock climbing (bouldering) as a new treatment for depression	2015	47 (22 persons in the intervention group, 25 persons in the waitlist group)	Patients with depression	8 weeks	1 × 3 h/week	24 h	Bouldering (wall up to 4 m)	Significantly higher improvement in the BDI-ll Score (Becks depression inventory) in the intervention group compared to the waitlist group
Aras and Ewert	The effects of 8 weeks sport rock climbing training on anxiety	2016	19 (9 persons in the intervention group, 10 persons in the control group)	Healthy sedentary adults	8 weeks	3 × 1 h/week	24 h	Sport climbing (wall up to 12 m)	Increased self-confidence and decreased somatic and cognitive anxiety in the intervention group
Schram Christensen et al.	To be active through indoor-climbing: an exploratory feasibility study in a group of children with cerebral palsy and typically developing children	2017	17 (11 children with and 6 without cerebral palsy)	11 to 13 years old children with cerebral palsy (GMFSC 1 and 2)	3 weeks	3 × 2.5 h/week	22.5 h	Sport climbing (wall up to 12 m) and bouldering	The children with cerebral palsy climbed a larger proportion of the route, children without cerebral palsy climbed faster. Improvement in the sit-to-stand test in children with cerebral palsy

## Results

### Physiology and impact of climbing

Despite an enormous knowledge about the positive effects of exercising on the human body “physical inactivity remains a pressing public health issue” (Haskell et al., 2007). Based on the energy supply, it is believed that climbing is composed of both aerobic and anaerobic elements. Depending on the angle of the wall and the difficulty of the climb, the contribution of the aerobic and anaerobic fractions may differ (Draper et al., 2010). Climbing “requires utilization of a significant portion of whole body aerobic capacity” (Sheel, 2004). Unlike many other sports, climbing combines different modalities of training, because it is a complex task for the motor system and coordination, involving balance, body stabilization and the simultaneous coordination of all four limbs (Stephan et al., 2011). Since climbing is a load-bearing strain, it results in a higher bone mass and an increased bone mineral content (Morrison and Schöffl, 2007). For preventing problems which come along with progressing age, climbing presents an opportunity to reduce the risk of osteoporosis and bone fracture.

Research has revealed that “climbing expends energy at a relatively high rate (…and) requires moderate levels of physical exertion and therefore may be a good activity for increasing cardiorespiratory fitness and muscular endurance” (Mermier et al., 1997). The heart rate increases of about 74–85% of the predicted maximum in indoor climbing (Mermier et al., 1997). It is interesting, that other than in most sports, the heart rate during climbing rises disproportionately in comparison to the surge of oxygen uptake. This can be explained by several circumstances: Firstly, it is known that isometric muscle contractions lead to this condition and that a great part of climbing is made up of this form of exercise. Secondly, the extent of cardiovascular response is proportional to the volume of included muscle mass which is extensive during climbing. Thirdly, the position of the arms plays an important role since they are mostly elevated above heart level and therefore contribute to a high heart rate (Mermier et al., 1997). By finding a significantly higher heart rate for both lead and top rope climbing just before the onset of the climb, a psychological involvement has been shown (Draper et al., 2010). Although increased psychological stress while climbing might explain a certain rise in heart rate more fully (Fleissner et al., 2010), data suggests that the rise in heart rate is primarily induced by physiological processes, whereas the psychological aspects make a smaller contribution (Sheel, 2004).

There is a high degree of planning and spatial orientation involved in climbing. The overall climbing-times differed significantly for the first and the subsequent attempts of the climbing route (Draper et al., 2008). By searching a viable and preferably the least straining path through a climbing route, the climber is forced to resort not only to reserves of strength, but also to coordinative and strategic skills. Through the combination of “dynamic gymnastic-like movements with static isometric position holds, explosive strength, stamina and intense isometric gripping function” (Morrison and Schöffl, 2007) and training of coordination and balance, climbing demands a lot of sporting skills besides muscle strength. Depending on the shape and the distance between the holds, sport climbing requires the body to take on a wide range of different body positions, while remaining in equilibrium. Additionally, fine motor skills are necessary to move hands and feet precisely so that a constant solid position of the body is warranted (Stephan et al., 2011). Because climbing results in improvement of balance and coordination of the extremities, it might be a potential therapeutic starting point in patients with neurological disease (Stephan et al., 2011). Going more into detail and looking at the operative mechanisms, it has been demonstrated that the act of climbing forces the climber to execute a conception of the climbing task, to build up a strong body tension and to apply coordinative capabilities. In consequence, more endurance, higher flexibility and a more complex movement pattern are possible (Fleissner et al., 2010). Another unique property of sport climbing is that it provides a potential of sensorial integration by involving different sorts of sensation. For instance, the multiple stimuli of seeing the hold, feeling the shape of the hold and ensuring one's own stability through shifting the body's center of gravity, allow an unconscious schooling of uniting outer and inner perceptions (Kern, 2010). The revised ability to react to external and internal sensations displays another possibility of how climbing can enhance activities of everyday life.

### Impact of climbing on muscular strength

Muscle-strengthening exercises have beneficial effect on the human body by stimulating bone formation and slowing down bone resorption and therefore reduce the risk of osteoporosis and fractures (Vuori, 2001). In addition, there seems to be a general relationship between higher muscular strength and lower overall-mortality (FitzGerald et al., 2004). Climbing appears to be well-suited to the promotion of muscular strength and trunk stability and mobility (Kim and Seo, 2015). This has been confirmed in a study which measured the effect of therapeutic climbing on dorsal muscle cross-activation in two patients (Mally et al., 2013).

### Effects of climbing on cognition

Increased physical exercise leads to an enhanced cognitive performance (Gomez-Pinilla and Hillman, 2013). Targeted training of coordination substantially influences synapse formation in the motor cortex and streamlines the neuronal connectivity (Rieckmann and Broocks, 2013). Different neuroprotective effects can be caused by physical activity. It can induce morphological and functional changes in all kinds of brain areas. For instance, work done in animal models suggests that exercising promotes the hippocampal neurogenesis through increased release of growth factors and a better microcirculation (Chieffi et al., 2017a). This is important to consider because the hippocampus is involved in neurogenesis even in a mature human brain. Therefore, physical activity can contribute enhancing lifetime cognitive performance (Chieffi et al., 2017b). An important neuroendocrinologic effect of sport seems to be the release of prolactin triggered through training. This promotes neuronal plasticity and induces proliferation of oligodendrocyte precursor cells that for their part contribute to myelination (Rieckmann and Broocks, 2013).

### Effects of climbing on emotion, self-confidence, and self-esteem

An important aspect potentially distinguishing climbing from other common sports is that it supports “not only the physical but also the cognitive and emotional resources of the individual” (Luttenberger et al., 2015). In the most general sense, this means that sport climbing provides the facility to experience feelings of success and to increase self-confidence and self-esteem by allowing the participant to set a clear goal—for example one specific hold or the top of the route—and by inducing the sense of having mastered a difficult challenge (Kern, 2010; Buechter and Fechtelpeter, 2011). Although there is only a small risk of severe downfall in top-roping, success in rock climbing is closely linked to one's personal willingness to take a certain degree of risk. By overcoming fears of falling and reaching the top of a route, an increased sense of self-efficacy can result. While deciding between the determination to finish the climb and the possibility of a fall, the skills of mental and physical perception can be improved (Aras and Ewert, 2016). Positive effects on mental health induced by sporting activity have previously been observed. For example, exercising leads to the plain opportunity of gaining distance from stressful issues of the daily routine (The Research File, 1994). Regular physical exercise is inversely related to the outcome of diverse diseases involving physical as well as mental illnesses (Kesaniemi et al., 2001; Table 3). Climbing conveys long-term psychological modifications (Mally et al., 2013). It is well-known that physical exercise has an influence on mood and mental well-being. It seems to show an even greater effect if it is carried out regularly and in groups and if it is supervised. By performing exercises that are schooling coordination, an enhancement in specific cognitive abilities like concentration can be attained. Climbing affords an ability to combine several important aspects: firstly, the difficulty can be varied through different size, shape and number of holds and footholds. Secondly, climbing demands a lot of body control, coordination skills, and concentration. In addition, it is linked with acute emotions such as feelings of pride, fear, and trust (Luttenberger et al., 2015). In consideration of these criteria, climbing appears to be a very useful means of counteracting impairments in psychological health. Generally, it can contribute to a more balanced mood, leading to an improved quality of life. The changes in mood and quality of life are highly subjective and differ heavily depending on the individual. By using self-reported outcome criteria, Mehl and Wolf (2008) have determined the effect of a high wire intervention in persons with psychiatric diseases. The findings indicate a gain of affirmative emotional experience as well as a decreased level of depression. Compared to a control group which had received the same treatment except the high wire intervention, an improvement in self-reported quality of life was reported (Mehl and Wolf, 2008). Even though this study did not investigate climbing, there are similarities between high wire and climbing such as height, a great demand of concentration and coordination and related emotional stimuli. Therefore, it can be speculated that these effects might also occur in individuals who perform climbing.

**Table 3:** Positive and negative aspects of physical exercise on the human body.

**Positive aspects of sports**	**Negative aspects of sports**
Decreased risk of cardiovascular death (Haskell et al., 2007)	Time-consuming
Decreased risk of chronic diseases (Paoli and Bianco, 2015)	Cost (in example equipment, gym-course)
Increased feeling of well-being (The Research File, 1994)	Necessity to overcome convenience
Enhanced cognitive performance (Rieckmann and Broocks, 2013)	
Decreased risk of infection (Reimers et al., 2013)	
Prevention of obesity (Haskell et al., 2007)	
Improved information progressing (Voelcker-Rehage et al., 2011)	
Promotion of neuronal plasticity (Rieckmann and Broocks, 2013)	
Reduced risk of osteoporosis/fractures (Vuori, 2001)	

### Climbing and social interaction

A special form of social contact develops in sport climbing: the belayer and the climber form one team and need to rely completely on each other. Accordingly, people should take responsibility for each other and must reveal a great degree of trust in the partner (Buechter and Fechtelpeter, 2011). This may result in an improved ability of social interaction in daily life. Based on the experience of complete faith in another person's capability of assuring ones' safety and the feeling of appreciation through the partner by given the charge for his safety the other way around, a strong bond forms between two persons. By transferring these competencies to other areas of life, an upgrade of quality of life can be accomplished.

### Risk of sport climbing

In public, climbing is often thought of being a dangerous sport harboring a high risk of injury. There is converse data indicating that climbing appears to be a relatively safe sport by exhibiting an overall injury rate of only 0.002 injuries per 1,000 h of climbing activities (Schöffl et al., 2013). Moreover, injury statistics show a lower rate of injury in sport climbing compared to other types of sport like for example soccer and handball (Aras and Ewert, 2016). The reasons therefore are numerous. Different styles of climbing exist, each of which are characterized by different features. Bouldering means climbing in jump altitude and is usually secured by using mats which lie underneath the climber. Lead climbing describes the style of ascend where the climber takes the rope from the ground to the top of the route by making use of intermediate belay points that are affixed in regular intervals at the wall. Finally, top rope climbing represents a very safe style of climbing. While doing so, the climber uses a rope which is already attached at the end of the route. In this way, the risk of long falls is reduced to a minimum. Another vital piece of knowledge is the fact that, apart from the experience that climbing can be performed outdoors on rocks, modern climbing gyms provide the opportunity to exercise indoors and ensure a high degree of safety standard. As anticipated, the injury rate in climbing depends on the environment and the style of ascent. Hence, the risk of injury can be reduced by using indoor climbing walls and by performing top rope climbing rather than lead climbing (Schöffl et al., 2013). The low injury rate is also influenced by the climber's ability to reduce possible risky situations through appropriate behavior (Limb, 1995). While climbing, the possible risks seem more apparent than in other sports. Thus, the executer can brace her or himself by precisely checking the equipment, picking a route that complies with personal skills, choosing a reliable partner and making plans of how to attempt the climb. Since the risk of traumatic injuries while climbing can be reduced by using current safety standards, overuse syndromes are the most common injuries in indoor climbing. They make up about 80% of all registered injuries and usually affect the upper limb, particularly the fingers (Wright et al., 2001). To lessen the number of accidents due to human failure, alpine associations have pronounced the advice of the so called “partner check:” by reciprocal reviewing of the knot and the right handling of the belay device before climbing, a significant number of mistakes leading to accidents are supposed to be avoided (Schöffl et al., 2013). Altogether, climbing seems to be a sport that can be performed without hesitation as far as certain safety regulations are respected and the intensity is not carried to excess.

### Impact of sport climbing on various neurological and psychiatric disease conditions

In the assessed ten studies in various neurological and psychiatric disorders (Table 2) the duration of studies lasted between 3 and 10 weeks and contained a total climbing time between 2.5 and 24 h. Also, the number of participants showed a broad range between 4 and 247 persons. In addition, the investigated outcome parameters differed a lot.

Stephan et al. (2011) investigated the impact of a 6 weeks lasting climbing intervention on cerebellar ataxia in four affected patients with different neurological diseases. Improvements in certain measures for movement quality such as velocity were observed. In patients with preexisting balance disturbance, the intervention increased the whole-body balance. Altogether, the study participants have even reported beneficial effects on movement quality in their daily lives (Stephan et al., 2011).

The outcomes of the study of Fleissner et al. (2010) point out the benefits of therapeutic climbing compared to conventional physiotherapy in geriatric patients. The mean age of the attendants was 81 years and the intervention took place subsequent to an acute care in the geriatric ward. The mobility as well as the independence of the participants in the experimental group was significantly better as compared to the control group which received standard treatment (Fleissner et al., 2010).

Kim and Seo (2015) investigated the different outcomes on health-related quality of life (HRQOL) and muscle activation on two groups of patients with lower back pain. One group participated in a month of therapeutic climbing, whereas the control group performed a lumbar stabilization program. In both subgroups, HRQOL and lumbar muscle activity assessed by surface electromyographic activities of the lumbar muscles were measured. There was a relatively higher increment of HRQOL in the climbing group. The growth of muscle activity of the erector spinae was higher in the lumbar stabilization group, but on the contrary gain of activity of the rectus abdominis and internal and external oblique muscles of the abdomen was inversely higher for the climbing group (Kim and Seo, 2015).

Patients with chronic lower back pain participated in a therapeutic climbing training that lasted 4 weeks and were compared to a control group that received standard exercise therapy (Engbert and Weber, 2011). The main outcome of the study was the physical and mental health of the participants measured by the SF-36 questionnaire. The results showed no significant difference between the intervention group and the control group. In three of eight scales, both groups showed an increase throughout the time of the study. In one scale solely, the control group and in two more scales solely, the intervention group improved (Engbert and Weber, 2011).

The assumption that climbing can be beneficial in patients with psychiatric diseases was confirmed by the findings of Luttenberger et al. (2015), which indicate that indoor climbing has a positive influence as an add-on therapy for depression. By using questionnaires, the authors compared a group of patients who underwent a climbing intervention lasting 8 weeks to a waitlist group without any additional measures. Eventually, there was a significant difference between both groups regarding depression scores in favor of the intervention therapy. The higher the symptom severity at onset of the intervention, the higher was the improvement in the outcomes (Luttenberger et al., 2015).

Aras and Ewert (2016) investigated the effect of an 8-week sport rock climbing training on anxiety by comparing an intervention group and a control group (Aras and Ewert, 2016). The term anxiety can be divided into two different aspects: “cognitive anxiety is the mental component of anxiety, and causes negative self-evaluations and doubts. Somatic anxiety includes physiological components and affects the organism directly” (Aras and Ewert, 2016). The study pointed to a decrease in cognitive and somatic anxiety as well as an increased level of self-confidence for the experimental group, whereas the control group failed to show a difference in any of these parameters (Aras and Ewert, 2016).

Children with cerebral palsy and controls took part in a study investigating climbing as a therapeutic option (Schram Christensen et al., 2017). Interestingly, the children with cerebral palsy climbed a larger proportion of the route whereas the controls climbed faster. There was an improvement in the sit-to-stand test in children with cerebral palsy. The authors concluded that the improved motor abilities obtained through the training were due to increased synchronization between cerebral cortex and musculature. This resulted in more efficient motor unit recruitment with positive impact on daily functional abilities. Moreover, in another study, children with special needs of different kinds improved by an indoor climbing program in self-efficacy (Mazzoni et al., 2009).

### Impact of climbing on multiple sclerosis

Physical exercise has beneficial effects in patients with MS (Petajan and White, 1999; Kesselring and Beer, 2005; Motl and Pilutti, 2012; Table 1). In a systematic review the consequences of strength training in persons with Parkinson Disease (PD) and MS were assessed (Cruickshank et al., 2015). In MS, the strength training was capable to elicit improvements in fatigue, quality of life, functional capacity and muscle activity. Several studies point toward positive effects of exercise in patients with MS regarding different outcome parameters like cognition and walking (Rodgers et al., 1999; Oken et al., 2004; Sandroff et al., 2014; Davies et al., 2016; Zimmer et al., 2017).

Fatigue represents a prevailing symptom of MS and is often mentioned as one of the most restricting factors in a patient's life (Popp et al., 2017). It is one of the main causes of impaired quality of life among MS patients (Braley and Chervin, 2010). Although there had been a lot of research in the recent years regarding potential causes and treatment of fatigue, the comprehension remains vague (Braley and Chervin, 2010). To date, despite a variety of available therapies for MS, there is still no established treatment for fatigue (Braley and Chervin, 2010). Various studies on the impact of exercise therapy on fatigue have revealed a significant effect in favor of physical activity (Oken et al., 2004; Heine et al., 2015). Particularly endurance and balance training as well as yoga had significant effects on fatigue, whereas muscle power training showed no significant impact on its alleviation (Heine et al., 2015). Although 40–80% of the patients with MS show a so-called Uhthoff's phenomenon, which is described as a temporary worsening of neurologic symptoms caused by elevated body temperature during physical activity, more than half of it spontaneously regresses after a short period of training (Frevel and Mäurer, 2013).

A study of Velikonja et al. (2010) investigated the influence of sport climbing and yoga on spasticity, cognitive impairment, mood change, and fatigue in people with MS by comparing test scores raised previous and afterwards of the respective intervention (Table 2). Although there were no improvements regarding spasticity, executive cognitive function and mood in both groups, it is noteworthy that there was an effect of sport climbing on the level of fatigue in patients with MS. In these patients the impact of fatigue on cognitive and on physical functions was reduced. Even though there was no significant impact of reduced fatigue on psychosocial functions, it is important to mention that the authors observed a growing group dynamic during climbing sessions which seemed to be a crucial motivational factor. Therefore, it can be assumed that sport climbing might have a potential to diminish fatigue in everyday life (Velikonja et al., 2010).

Obesity seems to be a risk factor in young girls to develop clinically isolated syndrome (CIS) and MS (Langer-Gould et al., 2013). Possibly exercise such as sport climbing can reduce the risk of developing CIS and MS.

Experimental autoimmune encephalomyelitis (EAE) is the animal model of MS that can be actively induced by immunization strategies with myelin proteins or peptides in adjuvants or by passive transfer of T cells (Weissert, 2012). The neuropathological effects of exercise in EAE were assessed by comparing mice that used a running wheel compared to sedentary mice (Pryor et al., 2015). The sedentary mice showed a significant higher mean disease score determined by behavioral testing. In addition, the disease onset after EAE induction was significantly later in the group with the running wheel. Another interesting result of this study shows a difference in demyelination between the two groups of mice, which was more distinct in the sedentary group. A higher density of axons in the ventral medial spinal cord and a significant greater number of motor neurons in the mice using the running wheel was observed. Taken together this study provides evidence that exercise in EAE is an effective way to attenuate symptom severity and to defer the disease onset (Pryor et al., 2015).

In summary, it can be stated that, additional to general health effects, physical activity in people with MS initiates a great variety of further events (Petajan and White, 1999; Kesselring and Beer, 2005; Motl and Pilutti, 2012; Table 4). To name a few, it can confine functional constraint, retain range of motion, forestall functional loss and enhance well-being (Frevel and Mäurer, 2013). The major advantage of exercise therapy is that it is a “relatively simple, easy accessible, non-invasive intervention” (Heine et al., 2015). For this reason, its effectiveness and because its execution is proven to be safe, it should be contemplated as a therapeutic starting point supplementary to the common concept of therapy. Altogether, the effects induced through climbing appear to be multilayered and obviously affect the physiological as well as the psychological capabilities of an individual (Table 5).

**Table 4:** Effects of physical exercise on MS.

**Potentially positive effects of physical activity in patients with MS**	**Potentially negative effects of physical activity on patients with MS**
Confinement of functional constraint	Danger of sustaining a worsening of symptoms
Improvement of functional capacity	Possibility of demotivation through worsening of the disease
Elevated feeling of self-confidence and self-efficacy	Aggravated execution (depending on form of sport and characteristic of symptoms)
Reduction of spasticity	Increased pain
Reduction of fatigue	
Retention of range of motion	
Possible neuroprotective effects	
Improved dealing with symptoms	
Prevention of comorbidities	
Communication in groups	

**Table 5:** Positive and negative aspects of climbing.

**Positive aspects of sport climbing**	**Negative aspects of sport climbing**
Promotion of physical, cognitive, and emotional components	Cost-intensive (e.g., equipment, admission, journey)
Multidimensional training (e.g., strength, balance, coordination, concentration)	Need of suitable environment (climbing gym, rock)
High potential to increase self-efficacy	Need of qualified trainer
Enhanced self-perception	Possible restriction through acrophobia
Forming of unique social contact	
Achievable feasibility for everyone through easily modifiable difficulty	
High motivational aspect	
Relatively small risk of injury	

## Discussion

### Therapeutic potential of climbing

In summary, climbing has many positive influences on body and mind. Since it is built of various elements governing, among others, motor function, coordination skills, concentration and psychological aspects, climbing warrants a highly comprehensive training. In the light of the fact that not many other sports are able to supply such a profound impact on multiple aspects of health and sense of well-being, it is less surprising that this sport is thought to be helpful in the treatment and management of several neurological and psychiatric diseases. Climbing belongs to the most primordial forms of movement that are important in evolution (Zajetz, 2014). There are existing rudimentary programs encoded in the CNS for the regulation and conditioning of muscle groups. While climbing, three-dimensional patterns with alternating static and dynamic muscle activities are recalled. That is why by performing therapeutic climbing, especially persons suffering from neurological impairment may benefit from these holistic exercises involving many functional systems (Kern, 2010). This sport provides participants with “opportunities to observe their limits and strengths, both in psychological and physiological terms” (Aras and Ewert, 2016). Regarding all these aspects and the fact that climbing can be performed by almost everyone, because it is possible to vary the requisition individually by different levels of difficulty, it appears legitimate to integrate climbing into a therapeutic concept.

Based on our review, it can be stated that even if various studies approached the effects of climbing on different neurological and psychiatric health conditions, it is very difficult to make a definite statement about its impact. This is due to the great variety of examined outcome parameters, the small number of participants and an inadequate length of the studies respectively the total climbing time. Nevertheless, some of the studies indicate a promising potential for climbing as a therapeutic intervention in different health conditions. Moreover, data from disease models in rodents is very promising and points toward the great potential of this intervention.

Hence, the question arises why there are just a few programs that contain climbing as a part of a therapy. The reasons therefore are complex as indicated below. Undoubtedly, although climbing evolves more and more from an extraordinary sport toward a mass sport, it is still less popular than other sports. Therefore, probably neither the patients nor the treating physicians think about it as an option in therapy. If an exercise therapy is considered at all, the tendency goes toward sports such as aquatic training, Nordic walking or cycling. In addition, the image of climbing in the public might be partly distorted due to the unilateral media reports about this topic. In the media there is a bias toward reporting remarkable achievements in climbing, presenting it as an extreme sport or covering severe climbing accidents, but neglecting its assumed therapeutic effects. Therefore, many people think of climbing as an exhausting and very dangerous sport. Furthermore, climbing cannot be performed as easily as other sports. Apart from a reliable partner, an appropriate environment in the form of a climbing gym or a rock is necessary. Moreover, fundamental knowledge about the handling of the material is required. A qualified trainer is indispensable for introducing the beginner in the technique of belaying and climbing and pointing out the basic aspects to ensure safety. Especially when climbing is used as a part of a therapy, the trainer needs to establish a special training schedule which takes into consideration possible impairments of the participants caused by their disease. To mount a medically sensible training program as part of a therapy, a lot of preparation regarding the education of the trainer and the development of a detailed program are necessary. A close cooperation between the physician in charge, the trainer and the participating patients represents the basis of a useful intervention. Regarding working time and recreational activities, it seems obvious that most people do not achieve the postulated amount of at least 30 min of moderate intensity aerobic physical activity on 5 days each week that is recommended for the maintenance of a healthy lifestyle by the American College of Sports Medicine (ACSM) and the American Heart Association (AHA; Haskell et al., 2007). Even tor healthy persons, performing such an amount of sport is a difficult task. For those individuals suffering from impairments or diseases of any kind this effort is even more demanding. Nevertheless, it is necessary to use physical activity especially in such instances, because it can prevent further health problems, alleviate symptoms and work as complement to other therapies.

In a more and more sedentary way of life, it does not come as a surprise that there is a fast-increasing rate of obesity that also affects outcome of neurological and psychiatric diseases. Therefore, it is essential to integrate physical activity into the daily routine, because it is “the only discretionary component of daily energy expenditure” (Haskell et al., 2007). Even if the long-term effect of sports on weight loss is partially unknown, it is nevertheless a promising approach. Obesity is a well-known risk factor for cardiovascular and metabolic diseases. In an interesting study it is verified that a combination of a low-fat diet and moderate physical activity leads to a reduction of the body mass index (BMI) and changes the oxidative stress status and the lipid metabolism that play a role in the development of various diseases (Monda et al., 2014).

### Therapeutic climbing in MS

Up to now there is only little research about the subject how therapeutic climbing might influence MS. Nevertheless, the diverse symptoms of this disease suggest climbing to be suitable as a complementary therapeutic intervention in patients with MS (Velikonja et al., 2010). Beside general effects which come along with physical activity, climbing can result in an improvement in specific symptoms of MS like fatigue, cognitive deficits and spasticity (Giesser, 2015). In addition, other serious restrictions being part of MS like for example reduced balance and body stability might be antagonized. Also, the preservation and enlargement of strength achieved through climbing plays a vital role in the containment of MS and its symptoms. Ultimately, the psychological effects of climbing may be helpful for patients in coping with their disease and the limitations coming with it. Contrary to the former recommendation of refraining from physical activity to avoid worsening of MS symptoms, there are many arguments for exercising in MS (Petajan and White, 1999; Kesselring and Beer, 2005; Motl and Pilutti, 2012). Apart from the fact that exercise is mostly well-tolerated in patients with MS, it can be useful to prevent complications and the development of comorbidities and may even have neuroprotective influences as summarized above (Giesser, 2015).

### How should patients with MS perform sport climbing?

Persons with MS might be affected by the disease in very different ways, reaching from having no symptoms to being severely disabled. The participation in sports activities can be highly limited by the way and degree of the disease symptoms. Climbing can be varied in many ways to satisfy the needs of diverse individual necessities. For example, patients experiencing balance problems can benefit from a more firmly pull of the climbing rope. This would support the climber by taking some of the bodyweight. Paresis might be a symptom that compromises the execution of physical activity. In climbing, the difficulty can be varied by using a different number and different shapes of holds. The selection of climbing routes should be guided by the individual needs of the climber. Therefore, the distance between the holds can be reduced or enlarged and the size of the holds can be amplified to ensure a facilitated performance. Patients with MS suffering from visual impairments can also compete in a climbing program, because the visual function is only one of many domains that are addressed by climbing. A lack of the vision can be at least partly compensated by the sensory system. It is possible to climb a route without seeing the holds but only by feeling them. The difficulty can be adjusted by using a different quantity and varied sizes. In summary, climbing offers a lot of possibilities to be adapted for individual needs. Therefore, it can be conducted by persons with a very broad range of different kinds and severity of symptoms.

## Conclusion

To establish climbing as a common therapeutic approach, it will be necessary to spread the knowledge about existence and possible advantages of this sport amongst physicians, the general population and specifically patients with disabilities like patients with MS. To determine the therapeutic impact of a climbing intervention on MS, a multitude of further research is required. It is important to overcome the fear of climbing by informing the community and pointing to the high level of safety standard. Facilities and opportunities must be provided in which climbing can be individually tested under supervision to determine whether it is a suitable activity. In summary sport climbing offers a great potential for relieving symptoms and enhancing physical and mental fitness in patients with MS.

## Author contributions

JS and RW: Carried out the concept/design of the study, literature review, manuscript preparation, critical revision of the manuscript, and approval of the article.

### Conflict of interest statement

The authors declare that the research was conducted in the absence of any commercial or financial relationships that could be construed as a potential conflict of interest.

## Abbreviations

BMI: body mass index
CIS: clinically isolated syndrome
CNS: central nervous system
EAE: experimental autoimmune encephalomyelitis
MRI: magnetic resonance imaging
MS: multiple sclerosis
PD: Parkinson's disease
PPMS: primary progressive multiple sclerosis
RRMS: relapsing remitting multiple sclerosis
SPMS: secondary progressive multiple sclerosis.
